# DP4-Assisted Structure Elucidation of Isodemethylchodatin, a New Norlichexanthone Derivative Meager in H-Atoms, from the Lichen *Parmotrema tsavoense*

**DOI:** 10.3390/molecules24081527

**Published:** 2019-04-18

**Authors:** Thuc-Huy Duong, Mehdi A. Beniddir, Joël Boustie, Kim-Phi-Phung Nguyen, Warinthorn Chavasiri, Guillaume Bernadat, Pierre Le Pogam

**Affiliations:** 1 Department for Management of Science and Technology Development, Ton Duc Thang University, Ho Chi Minh City 748355, Vietnam; duongthuchuy@tdt.edu.vn; 2 Faculty of Applied Sciences, Ton Duc Thang University, Ho Chi Minh City 748355, Vietnam; 3 Équipe “Pharmacognosie–Chimie des Substances Naturelles”, BioCIS, Univ. Paris-Sud, CNRS, Université Paris-Saclay, 5 Rue Jean-Baptiste Clément, 92290 Châtenay-Malabry, France; mehdi.beniddir@u-psud.fr; 4 CNRS, ISCR (Institut des Sciences Chimiques de Rennes)–UMR 6226, Univ Rennes, F-35000 Rennes, France; joel.boustie@univ-rennes1.fr; 5 Department of Organic Chemistry, University of Science, National University–Ho Chi Minh City, 227 Nguyen Van Cu Str., Dist. 5, Ho Chi Minh City 748355, Vietnam; kimphiphung@yahoo.fr; 6 Natural Products Research Unit, Department of Chemistry, Faculty of Science, Chulalongkorn University, Phayathai Rd., Patumwan, Bangkok 10330, Thailand; warinthorn.c@chula.ac.th

**Keywords:** lichen, xanthone, norlichexanthone, *Parmotrema*, DFT-NMR

## Abstract

A phytochemical investigation of the foliose lichen *Parmotrema tsavoense* (Krog and Swinscow) Krog and Swinscow (Parmeliaceae) resulted in the isolation of a new trichlorinated xanthone, isodemethylchodatin. The structure elucidation of this new norlichexanthone derivative proved tricky owing to proton deficiency, and to the lack of NMR data of closely related analogues. The structure of this compound was determined based on an integrated interpretation of ^13^C-NMR chemical shifts, MS spectra, and DP4-based computational chemistry was also performed to provide an independent and unambiguous validation of the determined structure. Isodemethylchodatin represents the first chlorinated lichexanthone/norlichexanthone derivative bearing a methoxy group at C-5.

## 1. Introduction

Xanthones represent ubiquitous polyphenolic metabolites endowed with various and significant bioactivities [1]. This tricyclic scaffold exclusively arises from the polyketide pathway in fungi whereas it is of mixed biosynthetic origin (shikimate/polyketide) in plants, resulting in different substitution patterns [2]. These structural differences legitimate joint efforts on plants, fungi and microbes to obtain structurally diverse molecules displaying this privileged scaffold. Lichen xanthones, estimated to account for ca. 5% of the reported natural xanthones, can later be subdivided into two distinct series that are lichexanthone/norlichexanthone derivatives (displaying a canonical 1,3,6-trihydroxy-8-methylxanthone), while a rather limited number of lichen xanthones (essentially sustained by xanthone dimers), are related to ravenelin (i.e., 1,4,8-trihydroxy-3-methylxanthones) [2]. From these basic skeletons, the diversification of lichen xanthones mostly depends on (i) the degree and position of chlorination and (ii) the extent and positions of methylations of the phenolic groups [3]. As a consequence of these biosynthetic processes, lichen xanthones often result in proton-deficient structures that are tricky to elucidate owing to the scarce number of NMR signals and to multiple possible regioisomers, which often co-occur within a single lichen species, having formerly led to numerous erroneous assignments related to the low H/C ratio [4,5,6]. The reliability of structural assignments slowly increased over time as analytical approaches tailored to lichen xanthones arose such as standardized TLC procedures [7], and HPLC procedures based on their specific UV/Vis profile [8]. Likewise, structural assignments were later backed up by the total synthesis of most archetypal lichen xanthones [9,10,11]. Nevertheless, such procedures are tedious and rely on comparative identification rather than proper spectroscopic identification limiting the availability of complete ^1^H- and ^13^C-NMR datasets for lichen xanthones. This renders more difficult yet the elucidation of such new structures. Generally speaking, the structure elucidation of molecules presenting a severe deficit of proton is especially challenging as in such cases, “silent fragments” (i.e., deprived of hydrogen) prevent structure assembly based on HMBC correlations [12]. Accordingly, if the ratio of the number of protons to the sum of heavy atoms (e.g., C, N, O, S, Cl…) is below 2, it is widely admitted that structure elucidation can be difficult or may even be impossible based on sole NMR data and elemental composition information, following the so-called Crews rule [13]. As of 2019, new computational chemistry tools have emerged for confirmation of constitution in equivocal NMR-based assignments. For this purpose, the NMR chemical shifts can be calculated for all candidate structures (regioisomers or stereoisomers) through a Boltzmann-weighted average of the shifts for all low-energy conformers [14] and the so-called DP4 mathematical algorithm can later be used to determine which best fits the experimental data to determine the correct structure with quantifiable confidence [15]. This strategy proved to be reliable tools in natural products structure elucidation [16,17,18]. Within the frame of our continued interest in the phytochemical study of underinvestigated Vietnamese lichen species [19,20,21,22], an original trichlorinated and depauperate in proton norlichexanthone derivative, isodemethylchodatin, was isolated from the foliose lichen species *Parmotrema tsavoense*. We herein report on the isolation and structure elucidation of this new compound, jointly determined by the thorough interpretation of ^13^C-NMR data and ab initio methods based on Gauge-Independent Atomic Orbital (GIAO) methods.

## 2. Results and Discussion

Former chemical study of the ethyl acetate extract of *P. tsavoense* yielded a series of novel depsidones and diphenylethers, alongside known depsides, depsidones, paraconic acids and triterpenes [19]. The chemical investigation of the methanol extract recently afforded a series of structurally unique polyketides, the so-called tsavoenones A–C [22].

Compound **1** was isolated from the MeOH extract of the whole thallus of *P. tsavoense* by repetitive chromatographic separations based on silica gel column chromatography, size-exclusion chromatography on Sephadex LH-20 and preparative TLC.

Compound **1** was obtained as a yellow, amorphous solid with a molecular formula of C_15_H_9_O_6_Cl_3_, established by the HRESIMS ion at *m*/*z* 388.9393 [M − H]^−^ (calcd. for C_15_H_8_O_6_Cl_3_, 388.9392, Δ 0.26 ppm) with the mass spectrum displaying the characteristic envelope of signals for a trchlorinated molecule spanning from *m*/*z* 388.9 to 393.9. The UV spectrum with the maxima at 250 and 318 nm was evocative of a xanthone scaffold [8]. The ^1^H-NMR spectrum of **1** revealed one methyl group (δ_H_ 2.82, 3H, s), a methoxy group (δ_H_ 3.75, 3H, s), a broad hydroxy hydrogen signal at approximately δ_H_ 10.92 and a hydrogen-bonded hydroxy proton (δ_H_ 13.98, 1H, s). The ^13^C-NMR spectrum, in conjunction with the HSQC spectrum revealed the presence of a carbonyl (δ_C_ 179.6), ten tertiary (including oxygenated carbons at δ_C_ 154.1, 153.7, 147.4, and 131.4), three aromatic quaternary carbons (δ_C_ 135.9, 103.1, and 102.0). Collectively, these spectroscopic features defined compound **1** as a fully substituted trichlorinated xanthone. Up to now, only one such structure was reported, i.e., the isomeric demethylchodatin [23]. Unfortunately, this structure was elucidated by single-crystal X-ray crystallography analyses of its triacetate derivative and its ^13^C-NMR spectroscopic data were not reported. Owing to the scarce number of protons of this molecule (Crews score: 0.375), its structure elucidation was primarily based on the thorough interpretation of ^13^C-NMR chemical shifts, as anticipated based on incremented substituent effects. The long-range heteronuclear correlations from the methyl group at δ_H_ 2.82 and C-7 (δ_C_ 126.9), C-8 (δ_C_ 135.9) and C-8a (ca. δ_C_ 103.1) located this group at C-8. This deduction was further supported by the weak ^4^*J* coupling from CH_3_-8 to C-9 (δ_C_ 179.6). Owing to the fully substituted nature of **1**, a first chlorine atom must be located at C-7, the chemical shift of which indicated an oxygenated substituent to occur at C-6. The HMBC cross-peak of the protons at δ_H_ 3.75 to the carbon at δ_C_ 131.4 placed the methoxy group at the carbon resonating at δ_C_ 131.4. 

**Figure 1:**
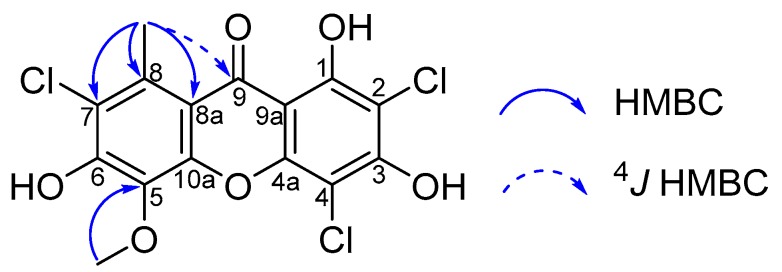
Complete set of HMBC correlations of **1**.

Such an upfield-shifted carbon resonance is not compatible with a C-6 location of this moiety. Indeed, the ^13^C-NMR spectroscopic data of unsubstituted xanthones showed that the chemical shifts of C-4/C-5 are 7–8 ppm upfield shifted compared to both C-1/C-8 and C-2/C-7, and 15 ppm upfield to C-3/C-6 [24,25]. Thus, monosubstituted xanthones having a C-4 methoxy group display a carbon resonance at δ_C_ 148.6 for this carbon (this substituent resulting in chemical shifts ranging from 155.7 to 165.0 ppm for the other substitution sites) [26]. Indeed, the chemical shift of the methoxy group-bearing carbon is diagnostic of its being placed at either C-4 or C-5 with a further shielding effect due to a hydroxy substituent, in excellent agreement with literature reports on such analogues [27,28,29,30]. Regarding the other nucleus, the hydrogen-bonded hydroxy group at δ_H_ 13.98 unambiguously established this phenol moiety at C-1. The chemical shift value of the carbon at δ_C_ 102.0 was diagnostic of a C-2 chlorinated carbon being flanked by two phenolic groups [3,10], consistently with the polyketide origin of lichen xanthones that leads to the lichexanthone-type 1,3-dioxygenated substitution pattern [2]. At last, the carbon resonance at δ_C_ 96.9 was highly evocative of a C-4 chlorinated carbon that is being shielded by both an ortho and a para hydroxy groups [26]. NMR data related to the right-hand ring were strongly supported by comparison to the ^13^C-NMR chemical shift values of 2,4-dichloronorlichexanthone that were in excellent agreement with the proposed attributions, that led us to surmise that the C-1 and C-3 chemical shifts might overlap (Table 1) [31]. A C-5 location of the third chlorine atom instead of C-4 would have resulted in the downfield shift of this carbon to values of approximately 107 ppm [3,10] due to the lack of the shielding effect of the para-disposed phenolic group [32]. Thus, the methoxy group must be linked at C-5 and not at C-4. Biosynthetic considerations would not support one of these candidate structures since the occurrence of an oxygenated substituent at either of these positions cannot be rationalized in regards to the polyketide origin of the norlichexanthone scaffold determined so far. A C-4 location instead of C-5 for this moiety would not have been consistent with its carbon resonating at δ_C_ 131.4 as the joint shielding effects from both an ortho and a para-hydroxy groups would have resulted in its shifting in the 126-128 ppm range [33,34,35,36]. NMR data related to unchlorinated xanthones displaying the same substitution pattern than the left-hand cycle could be compared to the determined constitution of **1**. A good agreement could be obtained with 5-*O*-methylated carbons being found to resonate ca. δ_C_ 133.0 [29,30]. At this stage, the chemical shifts of C-6 and C-10a were the last pending assignment. Such ^13^C-NMR chemical shifts can be expected to occur in a 152–156 ppm range [29,30], based on literature reports, indicating that these carbons might indeed correspond to either of the carbons found to resonate at δ_C_ 153.7 and/or 154.1 in our data set. This would account for the important intensity of the signal resonating at δ_C_ 153.7 despite its corresponding to tertiary oxygenated carbons. Collectively, these spectroscopic features would lead to determine compound **1** as the new structure depicted in Figure 1.

**Figure 2:**
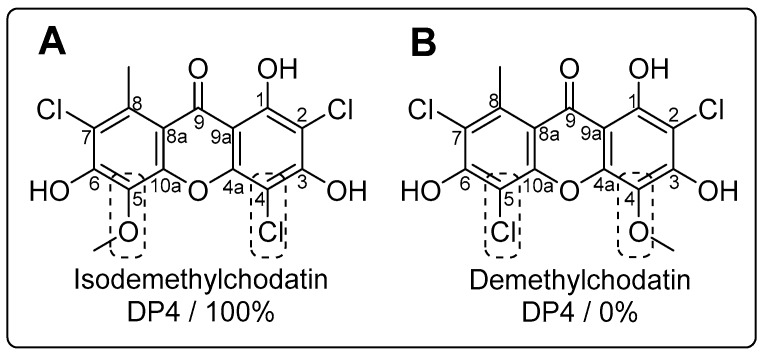
Chemical structures of the two possible regioisomers: tentative structure of isodemethylchodatin (**A**) and demethylchodatin (**B**) along with their respective DP4 probabilities.

To support this hypothesis, independent evidence were sought. Low-energy conformers of the two possible regioisomers, i.e., the preferred structure depicted in Figure 2A and demethylchodatin (Figure 2B) were determined and chemical shifts calculations using electronic structure methods of the lowest-energy conformers were analyzed with the DP4 probability method [15]. The comparison of ^13^C-NMR data of the two candidate regiosiomers with the observed chemical shifts of **1** through the DP4 probability method resulted in the prediction of the methoxy group being located at C-5 with a 100% probability. Thus, computational methods confirmed the original assignment of ^13^C-NMR spectra of **1**, independently of empirical chemical shift increments. In spite of the moderate B3LYP/6-31G(d) level of theory used, we were delighted to observe that accuracy of the predictions lied within 4 ppm in average (7 ppm in the worst case) to experimentally observed signals (Figure S8, Supplementary Materials). Altogether, these data validated the new structure of **1**, 2,4,7-trichloro-5-methoxynorlichexanthone or isodemethylchodatin, as depicted in Figure 1. The structural assignment of **1** based on its NMR data is fully supported by the comparison to the data of its non-chlorinated analogue, drimiopsin I [29]. The minute amount of this compound precluded the conducting of any biotest.

From a structural viewpoint, the description of new lichen xanthones having a monomeric and fully aromatized structure is getting increasingly unusual over time. For the last twenty years, only three research teams reported on such structures to the best of our knowledge. At first, a series of five xanthones isolated from aposymbiotically-cultured mycobionts of *Pyrenula japonica* and *P. bufonica* [37,38], a suite of prenylated xanthone heterosides from *Umbilicaria proboscidea* along with their acylated homologues [39,40], and later cladoxanthone A from *Cladonia incrassata* [41]. A salient feature regarding these structures is that none of them display a canonical substitution pattern relating them to either the norlichexanthone/lichexanthone or ravenelin subtype. The structural variations around these archetypal skeletons are limited given the scarce amount of modifications occurring on such scaffolds so it can reasonably be assumed that isodemethylchodatin stands among the last canonical norlichexanthone/lichexanthone-type derivative to be described. The biosynthetic origin of the 5-OCH_3_ group is not straightforward to delineate and is reminiscent of the 4-OCH_3_ moiety of demethylchodatin that is also difficult to account for. Noteworthily, 5-*O*-methylated norlichexanthones were recently isolated from non-lichenized fungi [29]. Yet, isodemethylchodatin represents the first occurrence of a chlorinated norlichexanthone/lichexanthone-type xanthone bearing a methoxy group at C-5. Isodemethylchodatin seems to be the second monomeric xanthone reported within Parmeliaceae lichens [42], besides lichexanthone which was reported in *Parmotrema lichexanthonicum* [43,44].

## 3. Materials and Methods

### 3.1. General

UV-Vis spectra were recorded as previously described [21]. NMR spectra were measured on an Avance III 500 MHz spectrometer (Bruker, Bremen, Germany) and the solvents residual signals were used as internal references (DMSO-*d*_6_, at *δ_H_* 2.50 and *δ_C_* 39.5 ppm). The ESI-HRMS data were recorded using a Bruker microTOF-Q II mass spectrometer (Bremen, Germany). Open column chromatographies were performed with silica gel 60 (40–63 µm, HiMedia, Mumbai, India) or Sephadex LH-20 (25–100 µm) (Pharmacia Fine Chemicals, Uppsala, Sweden). 

### 3.2. Lichen Material

References to the investigated lichen material were formerly provided elsewhere [22].

### 3.3. Extraction and Isolation

The chemical processing of the lichen material was performed as formerly reported [22]. Compound **1** (0.8 mg) was isolated from fraction P6.1.2 by thin-layer chromatography in a CHCl_3_/MeOH (88/12) solvent system.

*Isodemethylchodatin* (**1**). UV (EtOH) λ_max_ 250, 318 nm; ^1^H- and ^13^C-NMR see Table 1; HRESIMS *m*/*z* 388.9393 [M − H]^−^ (calcd for C_15_H_8_O_6_Cl_3_^−^, 388.9387).

### 3.4. Computational Chemistry

Conformations of compound **1** were fully optimized in vacuo and without constrain using DFT [45,46] with the hybrid Becke3LYP [47,48] functional and the 6-31G(d) basis [49], as implemented in the Gaussian 16 software package [50]. Upon geometrical optimization convergence, a frequency calculation within the harmonic approximation was conducted at the same level of theory and local minima were characterized by the absence of imaginary frequency. Chemical shifts were derived from NMR shielding tensors calculated using GIAO method [51,52] and corrected against values for the corresponding nucleus in TMS, both at the same level of theory. DP4 probability values were calculated using online implementation available from http://www-jmg.ch.cam.ac.uk/tools/nmr/DP4/.

## 4. Conclusions

Vietnamese lichen species are poorly studied from a chemical perspective, although recent studies from our group have shed light onto new scaffolds obtained from this untapped biota. A new trichlorinated norlichexanthone, isodemethylchodatin (**1**), was herein isolated from *Parmotrema tsavoense* and its structure was determined based on extensive spectroscopic data (^1^H-, ^13^C- and HMBC-NMR, HRMS). Owing to its fully substituted structure, the elucidation turned out to be quite challenging and was carried out by two independent approaches, i.e., (i) thorough interpretation of ^13^C-NMR data in regards to available NMR markers and substitution pattern-dependent predictable increments and (ii) Goodman probabilities. Besides representing the first occurrence of a canonical lichexanthone/norlichexanthone-type polyphenol being isolated from a lichen source since the compendium of lichen substances published by Huneck and Yoshimura in 1996, isodemethylchodatin represents the first occurrence of a chlorinated norlichexanthone/lichexanthone metabolite bearing an oxygenated substituent at C-5. Wider applications of DP4 to confirm constitution assigned by partly equivocal NMR data shall greatly secure the elucidation of such highly substituted structures that have led to many erroneous structural determinations throughout the last decades.

## Figures and Tables

**Table 1:** ^13^C- and ^1^H-NMR Spectroscopic Data (125/500 MHz) for **1** in DMSO-*d*_6_ (δ in ppm).

Position	*δ*_C_	*δ*_H_
1	153.7 ^1^	
2	102.0	
3	153.7 ^1^	
4	96.9	
4a	147.4	
5	131.4	
6	154.1 ^1^	
7	126.9	
8	135.9	
8a	103.1	
9	179.6	
9a	102.0	
10a	153.7 ^1^	
5-OCH_3_	60.5	3.76, *s*
8-CH_3_	19.2	2.82, *s*
1-OH		13.98, *br s*

^1^ Interchangeable signals.

## Supplementary Materials

The following data are available online. ^1^H-, ^13^C-NMR and HMBC spectra, HRMS spectra, Cartesian Coordinates (Ångstroms) and energies for **1** and demethylchodatin, DFT calculations results for Isodemethylchodatin **1** and Demethylchodatin, Parity plot of experimental and calculated ^13^C chemical shifts after linear regression with calculated ^13^C-NMR data of (A) Isodemethylchodatin (**1**) and (B) Demethylchodatin.
