# Supplementary material for: DP4-Assisted Structure Elucidation of Isodemethylchodatin, a New Norlichexanthone Derivative Meager in H-Atoms, from the Lichen Parmotrema tsavoense
Source: Molecules. 2019 Apr 18;24(8):1527. doi: 10.3390/molecules24081527 (PMC6515090; doi:10.3390/molecules24081527)

Supplementary Material Of

DP4-assisted Structure Elucidation of Isodemethylchodatin, A New Norlichexanthone Derivative Meager in H-atoms, From The Lichen *Parmotrema tsavoense*

Thuc-Huy Duong [1,2], Mehdi A. Beniddir [3], Joël Boustie [4], Kim-Phi-Phung Nguyen [5], Warinthorn Chavasiri [6], Guillaume Bernadat [3,*] and Pierre Le Pogam [3,*]

1 Department for Management of Science and Technology Development, Ton Duc Thang University, Ho Chi Minh City, Vietnam; duongthuchuy@tdt.edu.vn (T.D.H.)
2 Faculty of Applied Sciences, Ton Duc Thang University, Ho Chi Minh City, Vietnam.
3 Équipe "Pharmacognosie – Chimie des Substances Naturelles", BioCIS, Univ. Paris-Sud, CNRS, Université Paris-Saclay, 5 Rue Jean-Baptiste Clément, 92290, Châtenay-Malabry, France; mehdi.beniddir@u-psud.fr (M.A.B.); guillaume.bernadat@u-psud.fr (G.B.); pierre.le-pogam-alluard@u-psud.fr (P.L.P.)
4 Univ Rennes, CNRS, ISCR (Institut des Sciences Chimiques de Rennes) – UMR 6226, F-35000 Rennes, France; marylene.chollet@univ-rennes1.fr (M.C.K.); solenn.ferron@univ-rennes1.fr (S.F.); joel.boustie@univ-rennes1.fr (J.B.)
5 Department of Organic Chemistry, University of Science, National University – Ho Chi Minh City, 227 Nguyen Van Cu Str., Dist. 5, Ho Chi Minh City 748355, Vietnam; kimphiphung@yahoo.fr (K.P.P.N.)
6 Natural Products Research Unit, Department of Chemistry, Faculty of Science, Chulalongkorn University, Phayathai Rd., Patumwan, Bangkok 10330, Thailand; warinthorn.c@chula.ac.th (W.C.)

* Correspondence: guillaume.bernadat@u-psud.fr; +33-146-835-744 (G. B.); pierre.le-pogam-alluard@u-psud.fr; Tel.: +33-146-835-597 (P.L.P.)

Summary of the Supporting Material content

S1 HRESIMS of **1**

S2 ^{1}H-NMR spectrum of **1** (500 MHz, DMSO-d_6)

S3 ^{13}C-NMR spectrum of **1** (125 MHz, DMSO-d_6)

S4 HSQC spectrum of **1** (500/125 MHz, DMSO-d_6)

S5 HMBC spectrum of **1** (500/125 MHz, DMSO-d_6)

S6 Cartesian Coordinates (Ångstroms) and energies of Isodemethylchodatin **1**.

S7 Cartesian Coordinates (Ångstroms) and energies of Demethylchodatin

S8 DFT calculations results for Isodemethylchodatin **1** and Demethylchodatin and ^{13}C NMR Spectroscopic Data (125 MHz) for 1 in DMSO-d6 (δ in ppm)

S9. Parity plot of experimental and calculated ^{13}C chemical shifts after linear regression with calculated ^{13}C NMR data of (A) Isodemethylchodatin (**1**) and (B) Demethylchodatin

S1. HRESIMS of **1**

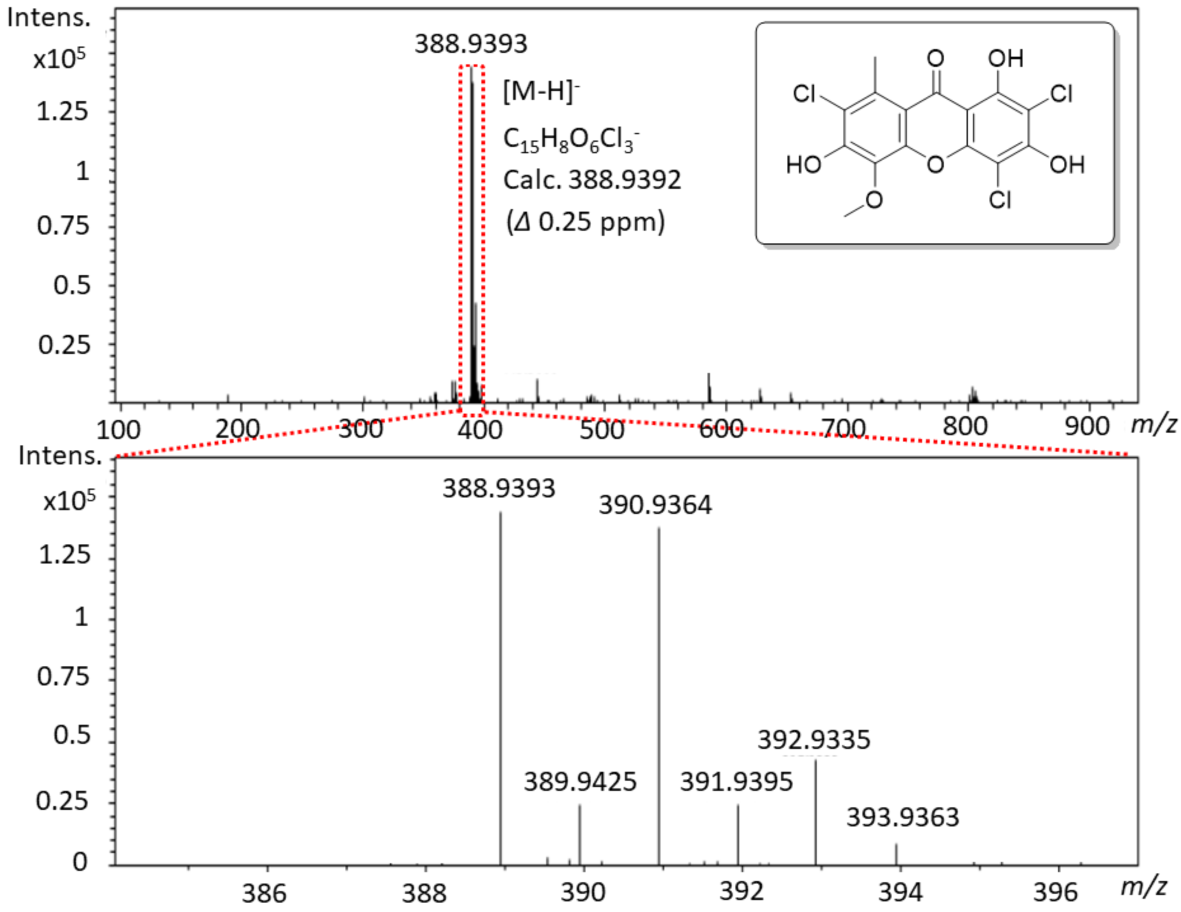

S2. ^{1}H-NMR spectrum of **1** (500 MHz, DMSO-d_6)

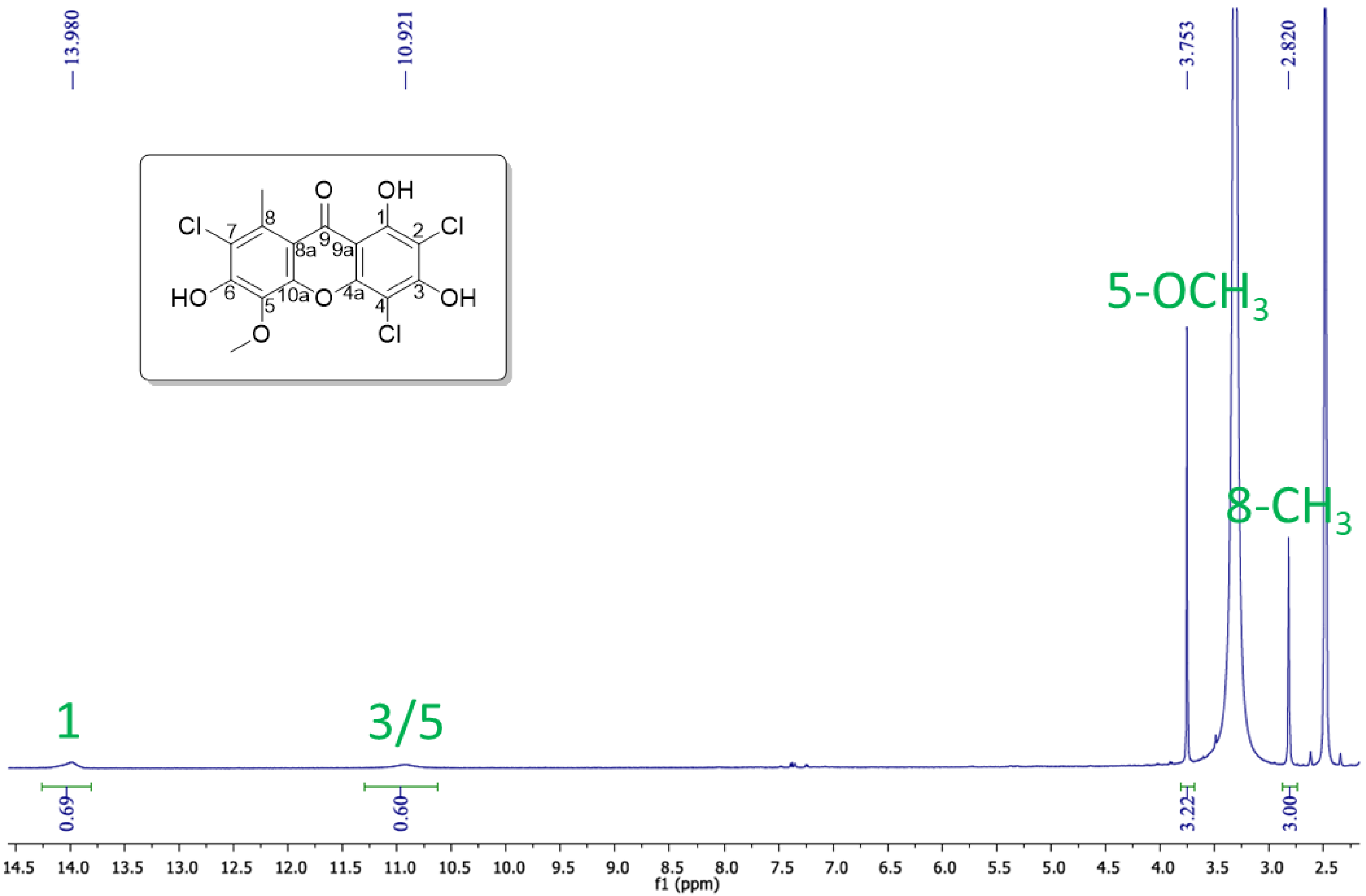

S3. ^{13}C-NMR spectrum of **1** (125 MHz, DMSO-*d₆*)

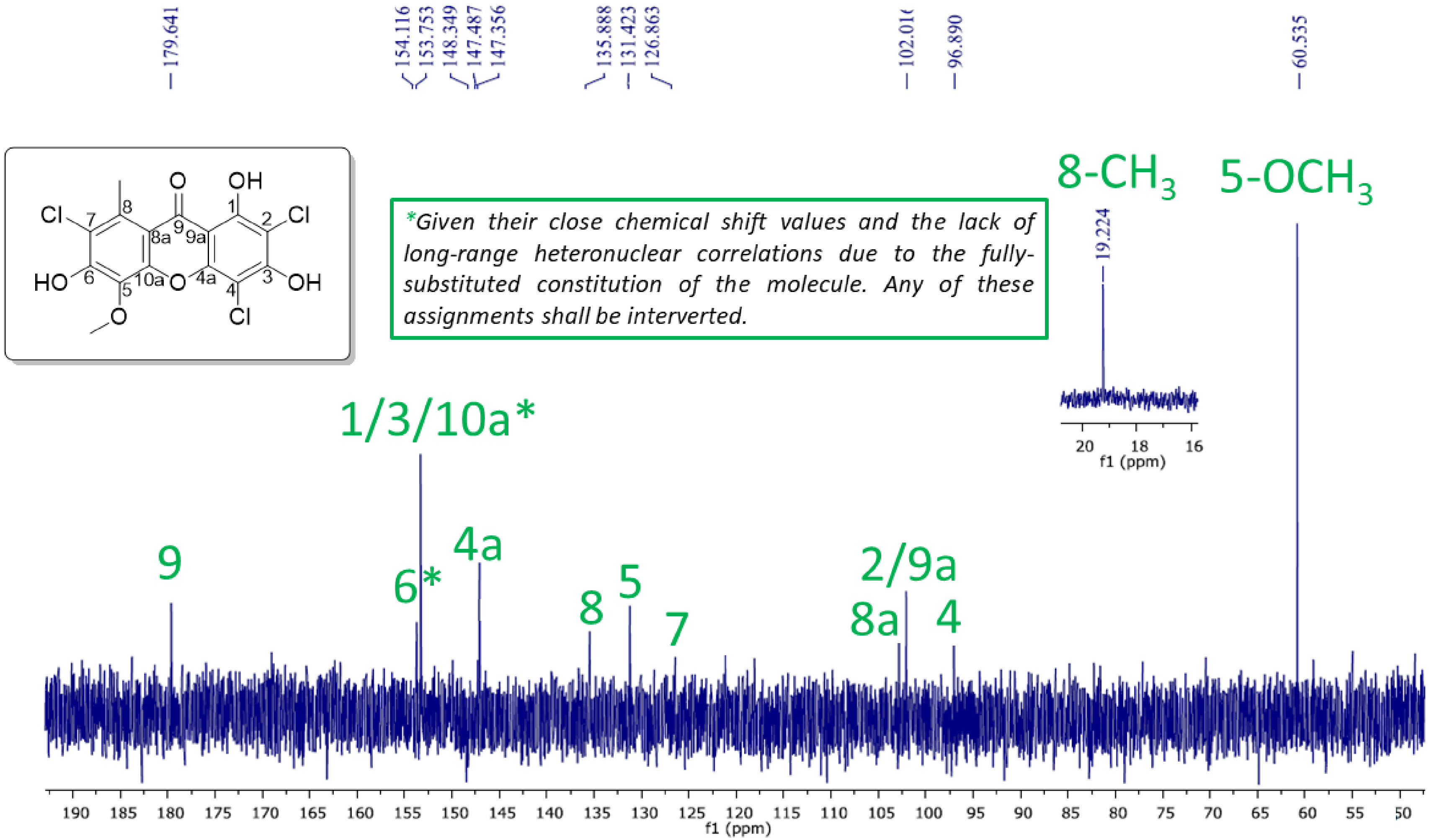

S4. HSQC spectrum of **1** (500/125 MHz, DMSO-d_6)

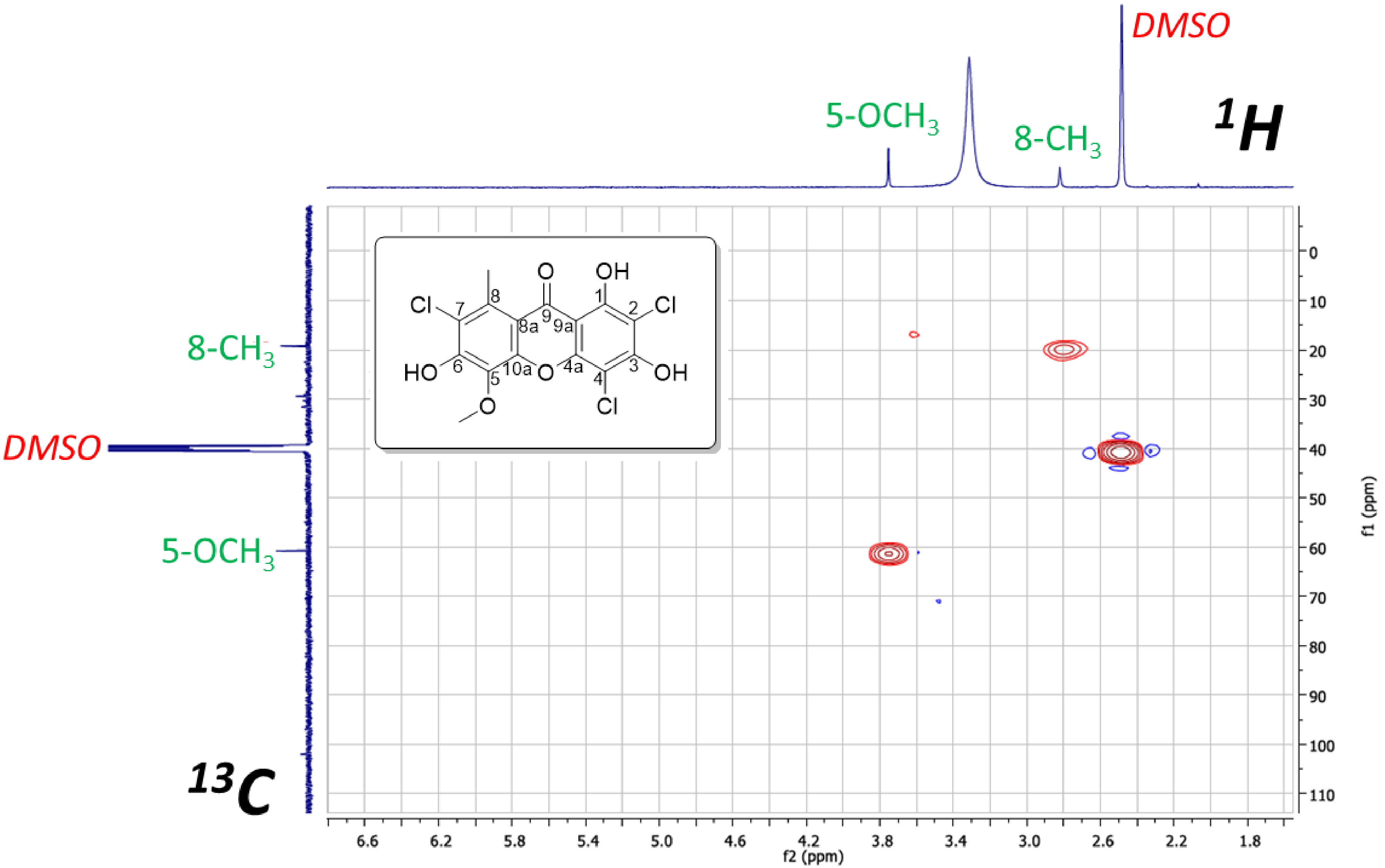

S5. HMBC spectrum of **1** (500/125 MHz, DMSO-*d*6)

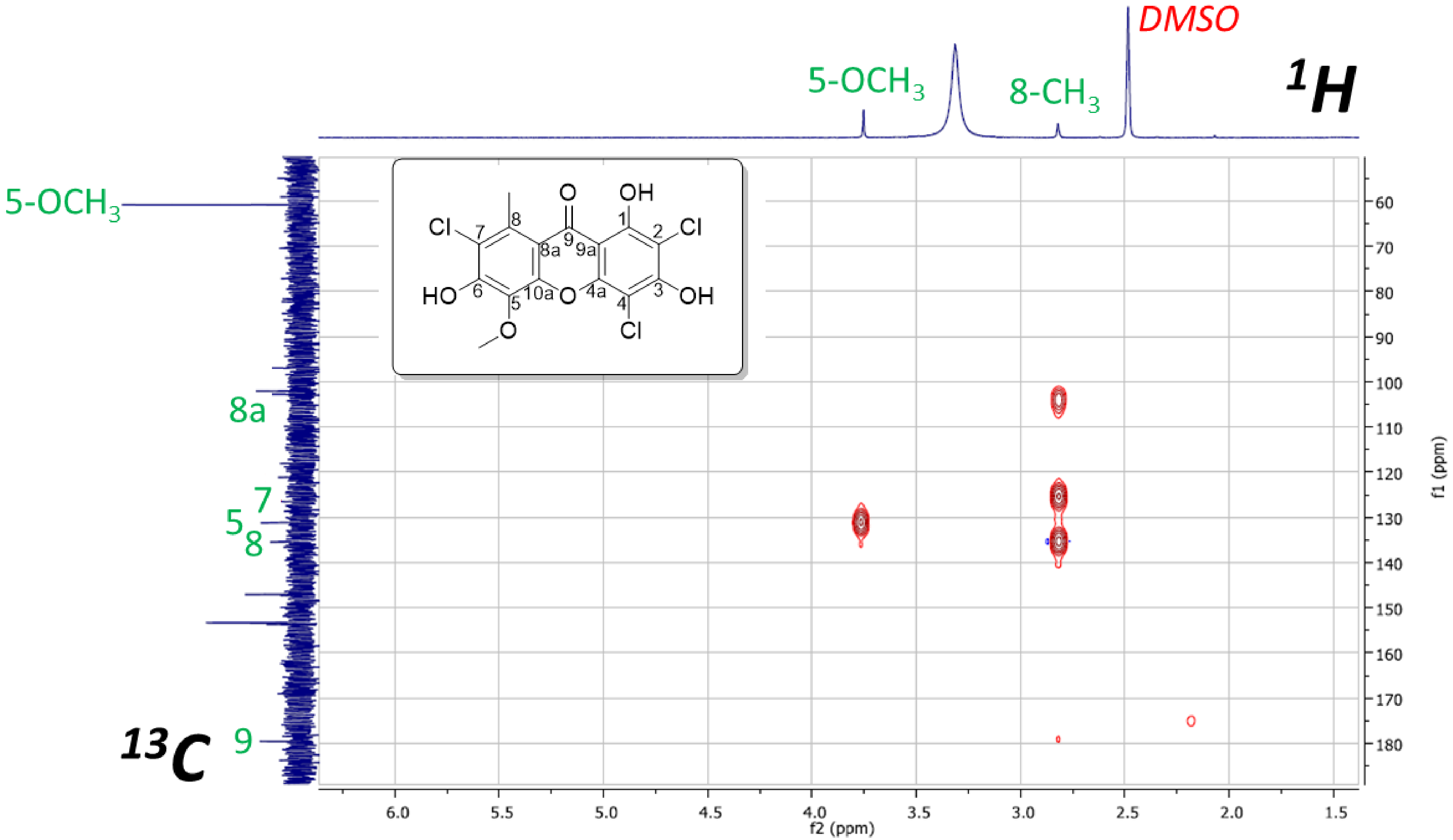

S6. Cartesian Coordinates (Ångstroms) and energies of Isodemethylchodatin **1**

E(B3LYP) = -2408.93616282 Ha, Lowest Frequency = 32.4656 cm^{-1}, Gibbs free energy = -2408.767101 Ha.

C	2.57039	-1.49985	0.00728
C	3.69564	-0.67470	-0.05676
C	3.58667	0.72795	-0.11681
C	2.32754	1.33495	-0.10794
C	1.18993	0.52975	-0.06990
C	1.28262	-0.87758	-0.00191
O	0.00471	1.20888	-0.10259
C	-1.17825	0.54400	-0.06493
C	-1.21198	-0.85834	0.00604
C	0.02488	-1.63526	0.04158
C	-2.34040	1.31310	-0.09739
C	-3.58652	0.66531	-0.06255
C	-3.63908	-0.73839	0.00664
C	-2.47751	-1.51405	0.04224
O	-0.01940	-2.88372	0.10547
O	-2.57604	-2.83909	0.10925
O	-4.69432	1.42349	-0.09567
Cl	-5.20743	-1.51810	0.04967
Cl	-2.26681	3.05114	-0.17922
O	2.31758	2.70555	-0.22374
C	1.76333	3.44325	0.88437
C	2.71640	-3.00094	0.07774
O	4.68648	1.49815	-0.18806
Cl	5.32873	-1.31518	-0.06358
H	-1.63441	-3.18410	0.12394
H	-5.47309	0.83729	-0.06691
H	2.00980	4.48932	0.69356
H	0.67913	3.32165	0.92700
H	2.21706	3.12112	1.82903
H	2.21816	-3.48143	-0.76843
H	3.76689	-3.28795	0.08138
H	2.23426	-3.39775	0.97483
H	4.36224	2.41680	-0.27463

S7. Cartesian Coordinates (Ångstroms) and energies of Demethylchodatin

E(B3LYP) = -2408.93403537 Ha, Lowest Frequency = 31.2373 cm^{-1}, Gibbs free energy = -2408.765064 Ha.

C	2.50174	-1.52385	0.04241
C	3.62904	-0.70557	0.00734
C	3.56470	0.70526	-0.06054
C	2.31116	1.31596	-0.09523
C	1.15938	0.52295	-0.06402
C	1.22614	-0.88469	0.00666
O	-0.01213	1.20950	-0.10539
C	-1.20889	0.55360	-0.07881
C	-1.26913	-0.84994	-0.00382
C	-0.04572	-1.63476	0.04099
C	-2.35730	1.33217	-0.11986
C	-3.61212	0.68977	-0.12151
C	-3.70871	-0.70622	-0.05516
C	-2.54759	-1.48751	0.00857
O	-0.08576	-2.88258	0.10629
O	-2.65823	-2.81434	0.07477
Cl	-5.27304	-1.46659	-0.05461
O	-4.72300	1.44321	-0.19425
O	-2.37739	2.70611	-0.24011
Cl	2.20009	3.04929	-0.17666
O	4.65334	1.49054	-0.09246
Cl	5.26700	-1.37629	0.04649
C	2.63429	-3.02597	0.11491
C	-1.88335	3.44273	0.89459
H	-1.72640	-3.17148	0.10391
H	-4.41210	2.36522	-0.29315
H	5.44857	0.92775	-0.06431
H	2.13707	-3.50023	-0.73469
H	3.68084	-3.32765	0.12860
H	2.13865	-3.41638	1.00717
H	-2.10981	4.49107	0.69029
H	-2.38881	3.12548	1.81463
H	-0.80311	3.31438	0.99872

S8. DFT calculations results for Isodemethylchodatin **1** and Demethylchodatin and ^{13}C NMR Spectroscopic Data (125 MHz) for **1** in DMSO-d_6 (δ in ppm)

Position	1	Demethylchodatin	Experimental Shifts
1	151.3	150.9	153.7[1]
2	104.2	105.9	102.0
3	146.9	147.5	153.7[1]
4	99.6	118.9	96.9
4a	144.0	140.1	147.4
5	125.6	109.2	131.4
6	151.3	150.9	154.1[1]
7	121.3	120.1	126.9
8	134.5	135.6	135.9
8a	108.7	109.9	103.1
9	172.8	172.4	179.6
9a	100.1	98.8	102.0
10a	151.3	150.9	153.7[1]
5-OCH_3	57.9	57.9	60.5
8-CH_3	20.4	21.3	19.2

[1] Interchangeable signals

S9. Parity plot of experimental and calculated ^{13}C chemical shifts after linear regression with calculated ^{13}C NMR data of (A) Isodemethylchodatin (**1**) and (B) Demethylchodatin

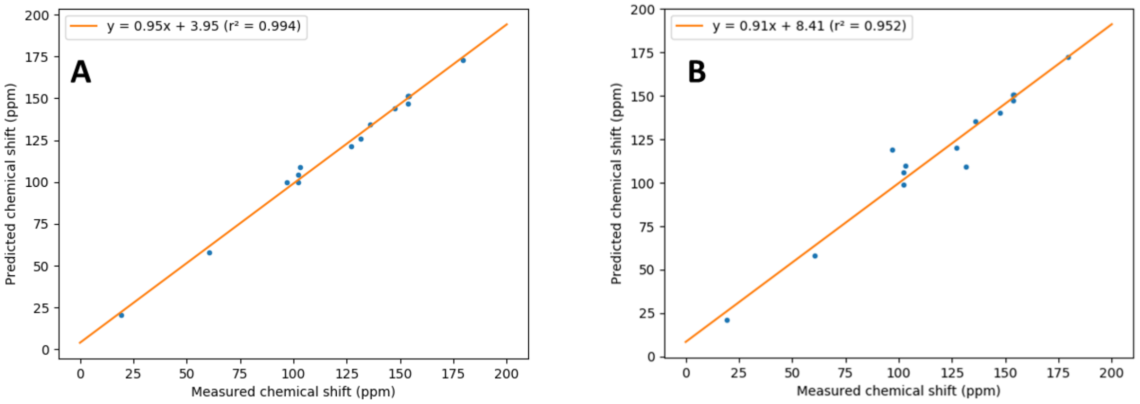

Supplement: Supplementary file 1 [file molecules-24-01527-s001.pdf]
